# Bartter Syndrome Type 3: Phenotype-Genotype Correlation and Favorable Response to Ibuprofen

**DOI:** 10.3389/fped.2018.00153

**Published:** 2018-05-30

**Authors:** Xuejun Yang, Gaofu Zhang, Mo Wang, Haiping Yang, Qiu Li

**Affiliations:** Department of Nephrology, Children‘s Hospital of Chongqing Medical University, Chongqing, China

**Keywords:** Bartter syndrome, hypokalemia, alkalosis, children, *CLCNKB*

## Abstract

**Objective:** To investigate the phenotype-genotype correlation in different genetic kinds of Bartter syndrome type 3 in children.

**Methods:** Clinical and genetic data of 2 patients with different mutations in Bartter syndrome type 3 was analyzed while the prognosis was compared after a 6-year follow-up or 2-year follow-up, respectively.

**Results:** Bartter syndrome is a kind of autosomal recessive inherited renal disorder. The manifestation and prognosis of Bartter syndrome change with mutation types, and severe mutation were often accompanied with unfavorable prognosis. Comprehensive therapy with ibuprofen, antisterone, captopril, and potassium have remarkable effect, while ibuprofen may improve growth retardation partly.

**Conclusion:** Bartter syndrome should be considered when children have unreasonable continuous electrolyte disturbance, metabolic alkalosis and growth retardation.As a genetic disease, its clinical features depend on the mutation type. It can be ameliorated by electrolyte supplementation, prostaglandin synthetase inhibitors, angiotensin-converting enzyme inhibitors and potassium-sparing diuretic. Considering the following electrolyte disturbances, infections, growth retardation, kidney failure and even death, Bartter syndrome need lifelong treatment, early diagnosis and treatment is the most important.

## Introduction

In this study, we reported two cases of Chinese patients with Bartter syndrome type 3 caused by different mutations of *CLCNKB*, which lead to different symptoms and different response to therapeutic measures (the written informed consent was obtained from the parents to publish the case report).

## Background

Bartter syndrome is a rare diseases, which results from congenital defects in the renal tubular system regulating the reabsorption of sodium, potassium and chloride (1). Based on the different pathogenic genes, Bartter syndrome was classified into five types (Type 1–5) (2). Meanwhile, classical Bartter syndrome, neonatal Bartter syndrome and variant Bartter syndrome (Gitelman syndrome) are the three clinical types of Bartter syndrome (3). Hypokalemia, hypochloremia, metabolic alkalosis, and growth retardation are the most common manifestations of Bartter syndrome (4), while the manifestation and prognosis change with mutation types, and patients with severe mutation often have a unfavorable prognosis. Moreover, comprehensive therapy with electrolyte supplementation, adequate fluid intake, prostaglandin synthetase inhibitors, angiotensin-converting enzyme inhibitors, and potassium-sparing diuretic have remarkable effects (4, 5).

## Case presentation

### Case 1

#### Clinical features

A 4-month-old female baby was sent to a children‘s hospital because of repeated vomiting and growth retardation. Her vomiting, which was not bilious or projectile, had lasted for 2 months and became more and more forceful recently. The baby looked thin and week, with a weight of 3.5 kg (≤3SD), a height of 54 cm (≤3SD), and a head circumference of 37 cm (≤3SD) (6).

The infant was born to a healthy 25-year-old G1P1 mother via spontaneous vaginal delivery at 38 weeks gestational age without antenatal polyhydramnios, with a birth weight of 2.9 kg and height 49 cm, and the Apgar scores was 9, 8, 10, at 1, 5, and 10 min, respectively. The patient‘s parents and relatives did not have any apparent clinical symptoms such as vomiting. There‘s no family history of consanguineous marriage and hereditary disease.

After admission, physical examination on the baby was performed. This revealed dehydration and delayed development, which manifested as disability of rising her head. Her blood pressure was 80/60 mmHg, pulse was 139 beats/min, and respiratory rate was 46/min. No rash, edema or hepatosplenomegaly was found. Circulatory, respiratory and neurologic examination did not reveal other specific deficit. Ultrasound of the gastrointestinal tract was normal, while ultrasound of the kidneys showed echo enhancement in both kidney, compatoible with nephrocalcinosis. Electrocadiography showed a low and flat T wave, accompanied with U wave. Serum electrolytes revealed hyponatremia, hypokalemia, and hypochloremia as follows: Na^+^ 122.5 mmol/l (normal range 135–145 mmol/l), K^+^ 1.8 mmol/l (normal range 3.5–5.5 mmol/l), Cl^−^ 56.6 mmol/l (normal range 95–110 mmol/l), Mg^2+^ 1.22 mmol/l (normal range 0.8–1.6 mmol/l), Ca^2+^ 2.57 mmol/l (normal range 2.15–2.75 mmol/l). Blood gas analysis showed metabolic alkalosis (pH 7.8, ${\text{HCO}}_{3}^{-}$ 35.7 mmol/L, pCO_2_ 5.6 Kpa). The serum aldosterone level was high (366 pg/ml, normal range 65–296 pg/ml), as well as the rennin activity (8.57 ng/ml/h, normal range 0.05–0.79 ng/ml/h), and the angiotensin II activity (1,084 pg/ml, normal range 28.2–52.5 pg/ml).

**Figure 1:**
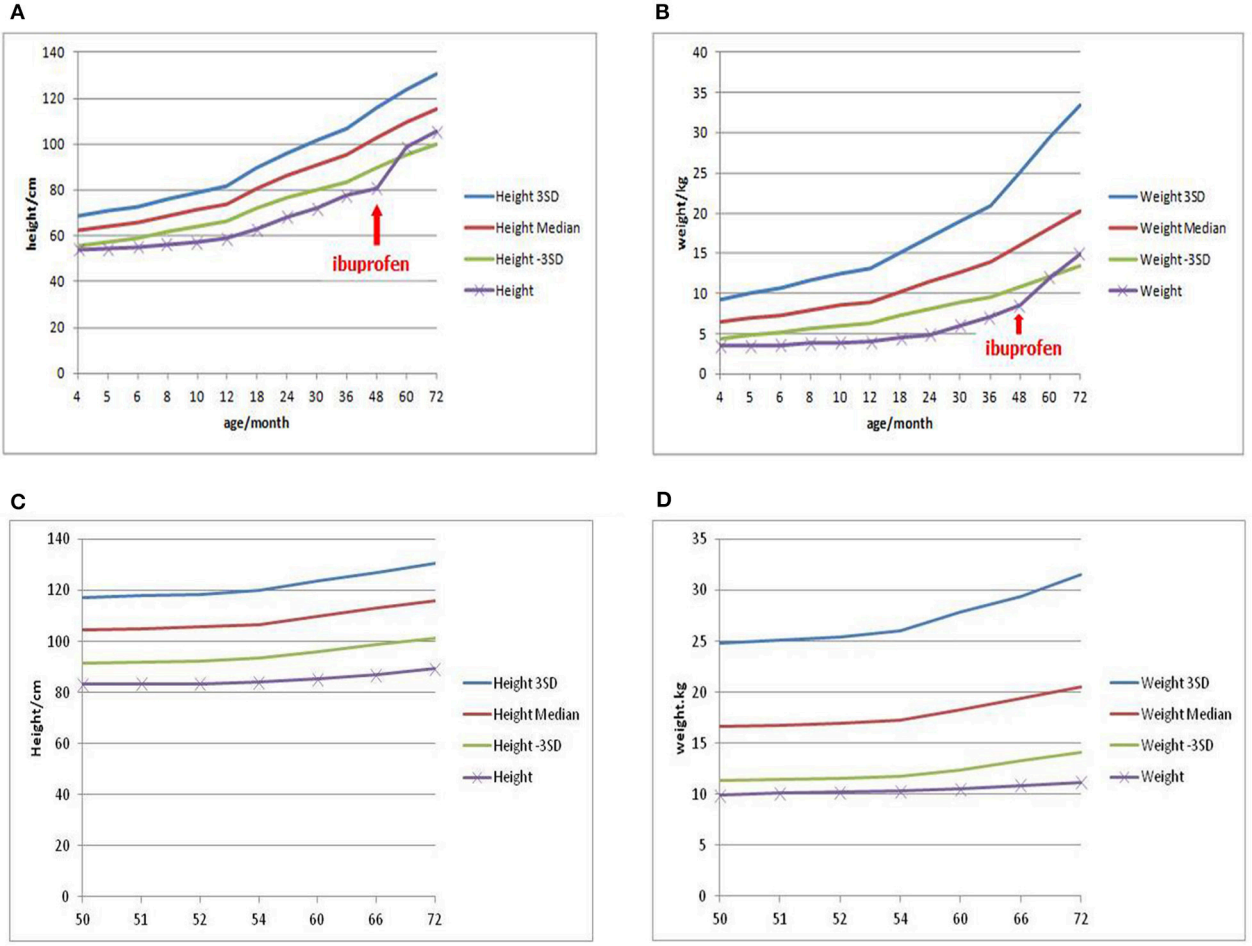
Growth curve of patient 1 showed the effect of ibuprofen in improving weight and height **(A,B)**, while Growth curve of patient 2 showed persistent growth retardation **(C,D)**.

In consideration of vomiting, growth retardation, hypokalemia, hypochloremia, and metabolic alkalosis, the infant was treated as a suspect case of Bartter syndrome on the second day. Spironolactone (1 mg/kg/d), catopril (1 mg/kg/d) for oral and adequate intravenous fluid therapy were given. Since the parents refused, prostaglandin synthetase inhibitors such as ibuprofen or indomethacin were not given at that time. On day 6, on account of the discontinued vomiting, normal serum electrolytes and blood gas analysis, the intravenous therapy was replaced of oral KCl solution (10 mmol/kg/d). On day 11, the baby was dismissed from hospital in-patient care with the therapy of KCl and increased fluid intake with age, then started a regular follow-up from then on. During the first 2 years, the baby did not vomit again. Serum electrolytes and blood gas analysis checked every month were normal. In the third year of follow up, when the girl was 4 years old, obvious growth retardation [weight 8.5 kg (≤3SD), height 75 cm (≤3SD)] was still observed (6). After a conversation with her parents, they agreed to start treatment with ibuprofen (30 mg/kg/d, 3 times a day). This led to improved length and wait gain in the following period. However, at the age of 6 years, the girl‘s weight was 14.9 kg (−3SD~−2SD) (Figure 1A), while the height was 105.4 cm (−2SD~−SD) (6) (Figure 1B).

#### Mutation analysis

Informed consent was obtained from the parents for mutational analysis of known Bartter syndrome genes. Genomic DNAs of the patients and their parents were extracted from peripheral blood, while DNA samples from 50 healthy unrelated Chinese people were severed as normal controls. Targeted sequencing using next-generation sequencing was conducted for genes responsible for Bartter syndrome (The detailed methods were in supplemental file 1).

**Figure 2:**
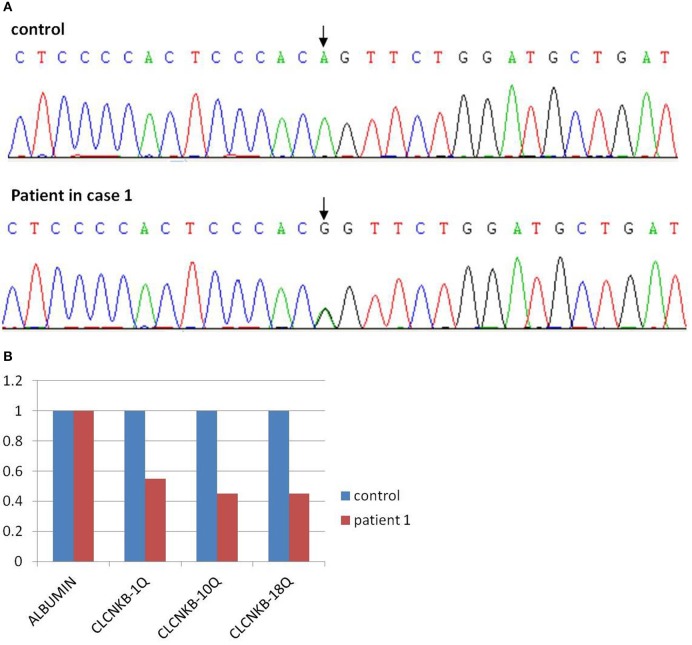
Mutation analysis of patient 1 showed a point mutation of CLCNKB **(A)** and a loss of exons 1–18 **(B)**.

As a result, two mutations of *CLCNKB* were identified. One is a homozygous transition (A–G) at the −2 position of the splicing acceptor site of intron 12 (NM_000085.4:C.1228-2A>G) from her mother (Figure 2A), which may resulted in the abnormal splice of exon 12. Another one is a heterozygous loss of exons 1–18(NM_000085.4: Ex1_18 del) from her father (Figure 2B). However, neither of these two mutations were detected in the control samples. Given the predicted devastating effect on protein structure of the 2 alleles, segregation within the family and no other mutations detected in known Bartter genes, we regarded the mutations as causative of Bartter syndrome type 3 (OMIM: 607364) in the baby.

### Case 2

#### Clinical features

A 4_2_/_12_-year-old boy was brought to hospital because of persistent hypokalemia and growth retardation. His serum potassium was 2.1 mmol/L the day before in a local hospital. He was born to a healthy 20-year-old G1P1 mother via spontaneous vaginal delivery at 39^+2^ weeks gestational age without antenatal polyhydramnios, with a birth weight of 3.4 kg and height 50 cm, and the Apgar scorea were normal. However, the patient‘s parents were first cousins without family history of hereditary disease.

On admisssion, his weight was 9.9 kg (≤3SD) and height was 83.2 cm (≤3SD) (6). His blood pressure was 92/58 mmHg, pulse was 101 beats/min, and respiratory rate was 31/min. Besides dental enamel dysplasia, no rash, edema or hepatosplenomegaly was found. No disorder showed in circulatory, respiratory, or neurologic examination. Ultrasound of the gastrointestinal tract and electrocadiography were normal while renal ultrasound examination showed echo enhancement in both kidney similar to what was observed in case 1 above. Serum electrolytes revealed hyponatremia, hypokalemia, and hypochloremia as follows: Na^+^ 111.9, K^+^ 2.3, Cl^−^ 70.3, Mg^2+^ 0.97, Ca^2+^ 2.52 mmol/L. Blood gas analysis showed metabolic alkalosis (pH 7.57, ${\text{HCO}}_{3}^{-}$ 39.4 mmol/L, pCO_2_ 5.73 Kpa). The serum aldosterone level was high (422 pg/ml), as well as the rennin activity (11.15 ng/ml/h), and the angiotensin II activity (1,459 pg/ml).

With the presentations of growth retardation, hypokalemia and metabolic alkalosis, the boy was clinically diagnosed as Bartter syndrome type 3. Spironolactone (1 mg/kg/d), catopril (1 mg/kg/d), ibuprofen (30 mg/kg/d) for oral and intravenous fluid therapy were given. On day 3, on account of the corrective serum electrolytes and blood gas analysis, the intravenous therapy was replaced by oral KCl solution (10 mmol/kg/d). On day 7, the boy left the hospital with the therapy of KCl and increased fluid intake with age, then started a regular follow-up by telephone from then on (Since he lived in a small village far from our hospital, his parents arranged him to attend a local clinic and informed us about the results via telephone). During these 2 years, serum electrolytes and blood gas analysis checked every month showed that hypokalemia, hypochloremia (K^+^ 2.8–4.0 mmol/L, Cl^−^ 84.1–100.5 mmol/L) and metabolic alkalosis (pH 7.37–7.58, ${\text{HCO}}_{3}^{-}$ 28.6–35.7 mmol/L, pCO_2_ 4.0–6.0 Kpa) still existed. At the age of six, the boy came back to us, still suffering from a severe growth retardation [weight 11.2 kg (≤3SD), height 89.4 cm (≤3SD)] (6) (Figures 1C,D), hypokalemia, hypochloremia (K^+^ 2.38 mmol/L, Cl^−^ 92.6 mmol/L) and metabolic alkalosis (pH 7.50, ${\text{HCO}}_{3}^{-}$ 32.3 mmol/L, pCO_2_ 5.9 Kpa).

#### Mutation analysis

**Figure 3:**
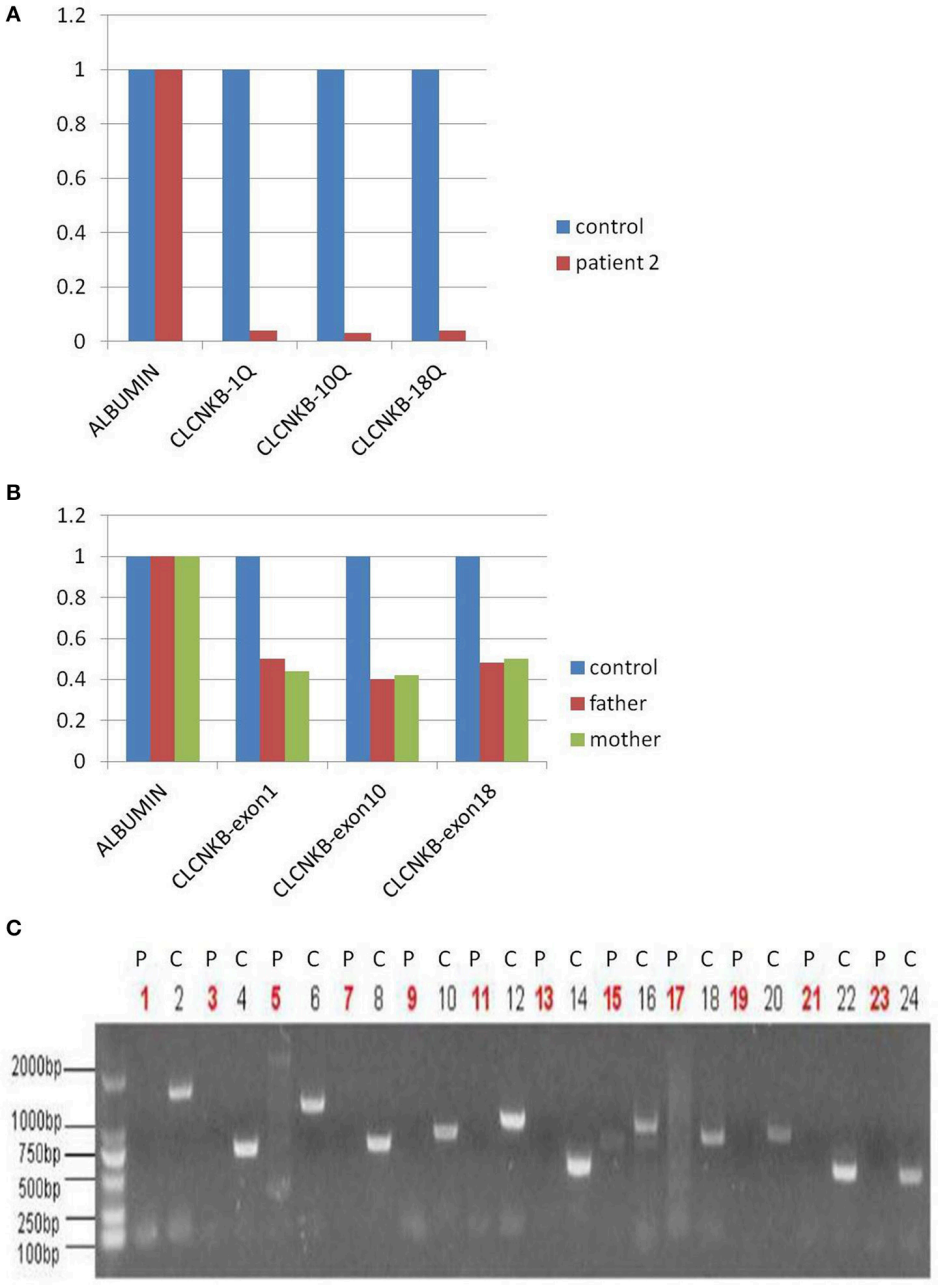
*CLCNKB* q-PCR analysis of patient 2 showed a homozygous loss of exons 1–18 in *CLCNKB* **(A)** while both parents appeared heterozygous **(B)**. Homozygous loss of *CLCNKB* is also shown by absence of bands in gel-electrophoresis of *CLCNKB* specific PCR products specific compared to a control sample **(C)**. P, patient; C, control.

In view of consanguineous marriage, NGS targeted sequencing of known Bartter syndrome genes and whole exome sequencing were both performed and revealed a mutation of CLCNKB. Unfortunately for the patient, since he descended from a consanguineous marriage, he inherited a large homozygous loss of exons 1–18 (NM_000085.4: Ex1_18 del) from his parents (Figures 3A,B), which was showed better by PCR electrophoresis of *CLCNKB* (Figure 3C). Meanwhile, another mutation of *FAM83H* was also identified, which was a homozygous transition (G to A) in exon 2 (NM_198488.2:C.154G>A), leading to amelogenesis imperfecta type 3 (OMIM: 130900).

## Discussion

Bartter syndrome autosomal-recessively inherited and characterized by the association of hypokalemia, hypochloremia, metabolic alkalosis, growth retardation and the activation of the renin-aldosterone axis (4). Since first reported by Bartter et al. (4), more and more patients have been diagnosed. However, a report in 2008 showed its incidence was about 1/1,00,000 (7). Based on the different underlying disease causing genes, Bartter syndrome was classified into five types with mutations in *SLC12A1, KCNJ1, CLCNKB, BSND*, and *CASR* identified to date (8) (Table 1).

**Table 1:** Genetic classification of Bartter syndrome.

**Type**	Inheritance^*^	**Gene**	**Affected Protein**
1	AR	SLC12A1	Sodium-potassium-chloride cotransporter (NKCC2)
2	AR	KCNJ1	Rectified potassium channel (ROMK)
3	AR	CLCNKB	Chloride channel Kb (ClC-Kb)
4	AR	BSND	Barttin protein
5	AD	CASR	Calcium-sensing receptor (CaSR)

* *AR, autosomal recessive inheritance; AD, autosomal dominant inheritance*.

Bartter syndrome type 3, caused by the mutation in *CLCNKB*, which encodes a protein called ClC-Kb. ClC-Kb is a member of the voltage dependent chloride channel family (ClC), which is expressed in the thick ascending limb of Henle‘s loop, distal tubule and cortical collecting tubule and regulates the tubular reabsorption of chloride in the kidney (9). As a result, mutations inactivate ClC-Kb, reducing chloride as well as sodium reabsorption in the renal tubules. Moreover, the loss of sodium chloride and water activates the renin-angiotensin-aldosterone system (RAAS), which contributes to the loss of potassium and renal fibrosis (10, 11). Up to now, more than 50 mutations of *CLCNKB* have been identified, most of which have unclear functional effect (12). In the present study, we identified two different CLNCKB mutations with one individual being compound heterozygous for a splice site mutations and deletion of the entrie gene and the seocnd case being homozygous for the deletion. None of varaints has been found in control samples. Brochard et al. (13) reported a similar *CLCNKB* gene splice mutation at the +1 position of the splicing acceptor site of intron 11 (NM_000085.4:C.1107+1G>T), which may lead to the loss of splice donor site. For another, according to Simon et al. (10), many patients have homozygous *CLCNKB* deletions which result in loss of normal gene function. In view of the foregoing and the patients' clinical features, we believe these mutations are pathogenetic.

As classical Bartter syndrome, Bartter syndrome type 3 is always accompanied with the mildest presentation, begins in infancy or later and often manifests with dehydration, electrolyte imbalance, polyuria, polydipsia, vomiting, and growth retardation (14). However, in both cases, we found echo enhancement in kidney, which may suggest nephrocalcinosis. Nephrocalcinosis and nephrolithiasis are usually found in antenatal and neonatal Bartter syndrome. Nevertheless, recently some studies have mentioned nephrocalcinosis in classical Bartter syndrome (15, 16). It is considered that *CLCNKB* mutations most commonly cause the classic Bartter phenotype, but in a minority of patients, they can also cause phenotypes that overlap with either antenatal Bartter syndrome/neonatal Bartter syndrome or Gitelman sydrome, such as nephrocalcinosis and nephrolithiasis. Exactly what causes nephrocalcinosis is unknown, but several factors may contribute to the condition, such as hypercalciuria, marginal hyperuricosuria, and hyperoxaluria (15).

For all types of Bartter syndrome, phenotypes depend on genotypes (17). Serious mutations always bring severe damage to patients, as in our present cases. Comprehensive therapy with electrolyte supplementation, adequate fluid intake, prostaglandin synthetase inhibitors, angiotensin-converting enzyme inhibitors and potassium-sparing diuretic have remarkable effect (5). Futhermore, for the past few years, more and more studies have raised that, on the basis of salt substitution, prostaglandin synthetase inhibitors may play an important part in improving growth retardation (18–20), which was also suggested in case 1. According to long-term follow-up studies in Bartter Syndrome, with appropriate treatment, patients with Bartter syndrome can achieve normal electrolyte values and growth parameters (21, 22), as we also observed in case 1 regarding the electrolytes while growth retardation remained. Case 2 however still showed electrolyte imbalance, alkalosis and growth retardation even after treatment with salt substitution and prostaglandin synthetase inhibitors. The large homozygous deletion of 18 exons of CLCNKB in case 2 may cause more severe loss of function than the compound heterozygous mutations in case 1. Secondly, considering the parental consangouinous marriage, case 2 may carry additional modifiying alleles such as the mentioned mutation of FAM83H, which may exacerbate his condition. Flyybjerg et al. (23) suggested that hypokalemia is a causative factor of growth retardation, while Masanori et al. (24) stated that sometimes classical Bartter syndrome may be complicated with growth hormone deficiency which could also be the case in case 2. Thus, more laboratory examinations such as growth hormone, whole genome sequencing and longer follow up will be necessary to establish the exact cause for reduced treatment response in Bartter Syndrome patients such as case 2.

In summary, we reported different prognosis of two Chinese patients with different *CLCNKB* gene mutations leading to Bartter syndrome type 3, as well as the effect of prostaglandin synthetase inhibitors in improving growth retardation. Since only two cases are limited in estimating the correlation between phenotype and genotype, more cases should be collected and analyzed in the future. However, considering the patients like which in case 2 still suffer from unsatisfied prognosis, some new treatment such as renin inhibitor (25), as well as more functional study of the mutations, examinations and long follow-up should be taken into account.

## Ethics statement

This research was approved by the Ethics review committee of Chlidren‘s Hospital of Chongqing Medical University. Since this is a case report, no protocol or ethics committee was utilized for this report. Any identifiable information has been removed from the manuscript.

## Author contributions

XY and GZ assumed clinical duties of this patient while he was hospitalized and drafted the initial manuscript. XY, HY, MW, and QL all reviewed and revised the manuscript. All authors approved the final case report as submitted and agree to be accountable for all aspects of the work.

### Conflict of interest statement

The authors declare that the research was conducted in the absence of any commercial or financial relationships that could be construed as a potential conflict of interest.
