# Supplementary material for: Bartter Syndrome Type 3: Phenotype-Genotype Correlation and Favorable Response to Ibuprofen
Source: Front Pediatr. 2018 May 30;6:153. doi: 10.3389/fped.2018.00153 (PMC5989644; doi:10.3389/fped.2018.00153)
Supplement: Supplementary file 1 [file Data_Sheet_1.docx]

Supplement 1: methods

DNA extraction

Genomic DNA was extracted from peripheral blood lymphocytes by standard procedures using QIAamp DNA Bloodmini kits (Qiagen, Germany). Next, 3 ug genomic DNA was fragmented by Covaris sonicator (Covaris S2, USA) to sizes of 150-300 bp and then purified.

Library Construction

The blunt ends of the purified DNA fragments were then repaired, and A-tailing was added. The fragments were ligated overnight using standard Illumina paired- end (PE) adapter. The ligated products were then amplified through 7-cycle polymerase chain reactions (PCRs) using PE primers containing 8 bp index tags.

Target region capture

The purified PCR products containing 3 ug DNA were hybridized to the GenCapTM probe solution (Mygenostics Co.Ltd.,China) at 65°C for 22 hours using a PCR thermocycler. The products were bound to a rotator for 1 hour at room temperature using Dynal Myone Streptavidin C1 magnetic beads (Invitrogen, USA), which had been activated beforehand, and the products were then washed with buffer according to the kit manual. The captured DNA libraries were amplified using 15-cycle PCRs, purified, and subsequently eluted in a 30 ul volume and subjected to Agilent 2100 Bioanalyzer and quantitative PCR to estimate the magnitude of enrichment.

Next Generation Sequencing

The final captured DNA libraries were sequenced using the Illumina HiSeq2500 DNA Sequencer as PE 90 bp reads (following the manufacturer’s standard cluster generation and sequencing protocols), providing an average coverage depth for each sample of at least 100-fold.

Data filtering and analysis

Image analysis, error estimation, and base calling were performed using the Illumina pipeline (version 1.3.4) with default parameters. Indexed primers were used to identify the different samples in the primary data. All unqualified reads (defined as reads either polluted by adapter, containing more than 10% nucleotides out of read length, having an average quality of less than 10, or having 50% bases with a quality value less than 5) were removed using a local dynamic programming algorithm. The remaining reads were aligned to the reference human genome (UCSC hg19) using Burrows-Wheeler Alignment Tool (BWA-0.7.12-r1044). Next, SNPs and indels were identified using GATK software3.4-46 using the recommended parameters.

Functional annotation of genetic variants

The variants were functionally annotated using an in-house pipeline as well as the reported frequencies available from public databases (dbSNP 144, HapMap database, ESP6500，EXAC，1000 genome variants database, and an internal database) and categorized into either missense, nonsense, splice-site, insertion, deletion, synonymous or noncoding mutations. For all variants, the results were filtered using a quality value of single base sequencing ≥ 20. The variants were filtered to potential mutation candidates through: 1). Functional variants (insertion/deletion: in CDS and splicing region, SNP: nonsense, splice site and missense) and 2). Variants with an allele frequency below 0.01 in either of the public databases mentioned above.

The identification of known pathogenic variants was based on mutations previously reported to cause Bartter syndrome and other diseases in the literature. Novel variants considered to be pathogenic were either: 1) stop/frameshift variants; 2) missense mutations positioned in the amino acid conservative region across species; 3) splice-site variations fulfilling the GT-AT rules; or 4) predicted to be possibly damaging or disease-causing by more than two of the bioinformatic programs (SIFT, Sorting Intolerant From Tolerant, http://sift.bii.a-star.edu.sg/; PolyPhen-2, http://genetics.bwh.harvard.edu/pph2/; Mutation Taster, http://www.mutationtaster.org/; BDGP, Berkeley Drosophila Genome Project, http://www.fruitfly.org/seq_tools/splice.html) .

Large deletions analysis

The depths of each region of a gene in different samples within the same sequencing lane are significantly correlated (r > 0.7), and the depth of each capture region was therefore used to calculate a z-score equation.

The large deletions were identified using a predefined cut-off point (±3) of derived z-score of each captured gene region. We used the cut-off value of 3 for absolute z- score, as it represents the 99.9th percentile of the normal samples set for one tailed region. Any region with a z-score above 3 was defined as a deletion (<-3).
